# Predictors of dopamine dysregulation syndrome in patients with early Parkinson’s disease

**DOI:** 10.1007/s10072-023-06956-w

**Published:** 2023-07-14

**Authors:** Keke Liang, Xiaohuan Li, Jianjun Ma, Hongqi Yang, Xiaoxue Shi, Yongyan Fan, Dawei Yang, Dashuai Guo, Chuanze Liu, Linrui Dong, Qingqing Chang, Qi GU, Siyuan Chen, Dongsheng Li

**Affiliations:** 1 Department of Neurology, Henan University People’s Hospital, Zhengzhou, China; 2 Department of Neurology, Henan Provincial People’s Hospital, Zhengzhou, China; 3 Department of Neurology, Zhengzhou University People’s Hospital, Zhengzhou, China

**Keywords:** Parkinson’s disease, Dopamine dysregulation syndrome, Impulse control disorder, Dopamine transporter, Risk factors

## Abstract

**Background:**

Dopamine dysregulation syndrome (DDS) is a complication of Parkinson’s disease (PD) that seriously affects the quality of life of PD patients. Currently, the risk factors for DDS are poorly known, and it is critical to identify them in the early stages of PD.

**Objective:**

To explore the incidence of and risk factors for DDS in patients with early PD.

**Methods:**

A retrospective cohort study was conducted on the general data, clinical features, and imaging data of patients with early PD in the PPMI database. Multivariate Cox regression analysis was performed to analyze the risk factors for the development of DDS in patients with early PD, and Kaplan‒Meier curves examined the frequency and predictors of incident DDS symptoms.

**Results:**

At baseline, 2.2% (*n* = 6) of patients with early PD developed DDS, and the cumulative incidence rates of DDS during the 5-year follow-up period were 2.8%, 6.4%, 10.8%, 15.5%, and 18.7%, respectively. In the multivariate Cox regression model controlling for age, sex, and drug use, hypersexuality (HR = 3.088; 95% CI: 1.416~6.732; *P* = 0.005), compulsive eating (HR = 3.299; 95% CI: 1.665~6.534; *P* = 0.001), compulsive shopping (HR = 3.899; 95% CI: 1.769~8.593; *P* = 0.001), anxiety (HR = 4.018; 95% CI: 2.136~7.599; *P* < 0.01), and lower Hoehn-Yahr (H-Y) stage (HR = 0.278; 95% CI: 0.152~0.509; *P* < 0.01) were independent risk factors for DDS in patients with early PD. PD patients with DDS had lower DAT uptake values than those patients without DDS.

**Conclusion:**

Early PD patients with hypersexuality, compulsive eating, compulsive shopping, anxiety, and lower H-Y stage were at increased risk for DDS. The occurrence of DDS may be related to the decrease in the average DAT uptake of the caudate and putamen.

## Introduction

Parkinson’s disease represents a fast-growing neurodegenerative condition [1], and its prevalence has been projected to double over the next 30 years [2]. The primary manifestations of PD are motor symptoms such as bradykinesia, resting tremor, and rigidity [3]. In addition to motor symptoms, PD patients often have nonmotor symptoms. such as cognitive impairment, neuropsychiatric symptoms, and sleep disorders.

Dopamine dysregulation syndrome (DDS) is a neuropsychiatric disorder in PD patients who abuse or become addicted to dopaminergic drugs. Patients with DDS self-administer increasing doses of dopaminergic drugs in excess of those required to control their symptoms or ask doctors for drug escalation. Even with severe drug-induced dyskinesias, they are still unwilling to stop taking the drugs, which seriously affects their quality of life [4]. Previous studies have shown that the incidence of DDS in PD patients is approximately 2.2–4% [5–9] and may be related to dysfunction of the dopaminergic reward system [10]. At present, there are few studies on DDS, and the risk factors for DDS in patients with PD are not clear. Indeed, while risk factors for DDS have been previously investigated [11, 12], to our knowledge, this study is the first to explore the risk factors for DDS in a cohort of untreated early PD patients who have been diagnosed via molecular imaging. This study also aims to further explore the relationship between striatal DAT uptake and DDS in PD patients to provide a basis for reducing its incidence.

## Materials and methods

### Research samples

The data used in this study were obtained from Parkinson’s Progression Markers Initiative (PPMI) database (www.ppmi-info.org/data). The PPMI is a 5-year observational, international, multicenter study sponsored by the Michael J. Fox Foundation in which subjects undergo clinical evaluations, imaging, and blood and cerebrospinal fluid collection at predetermined time points to explore biomarkers associated with PD progression. Each site participating in the PPMI was approved by the Ethics Standards Committee for Human Experimentation before the start of the study, and at each site, all subjects signed an informed consent form [13].

### Object selection

**Fig. 1:**
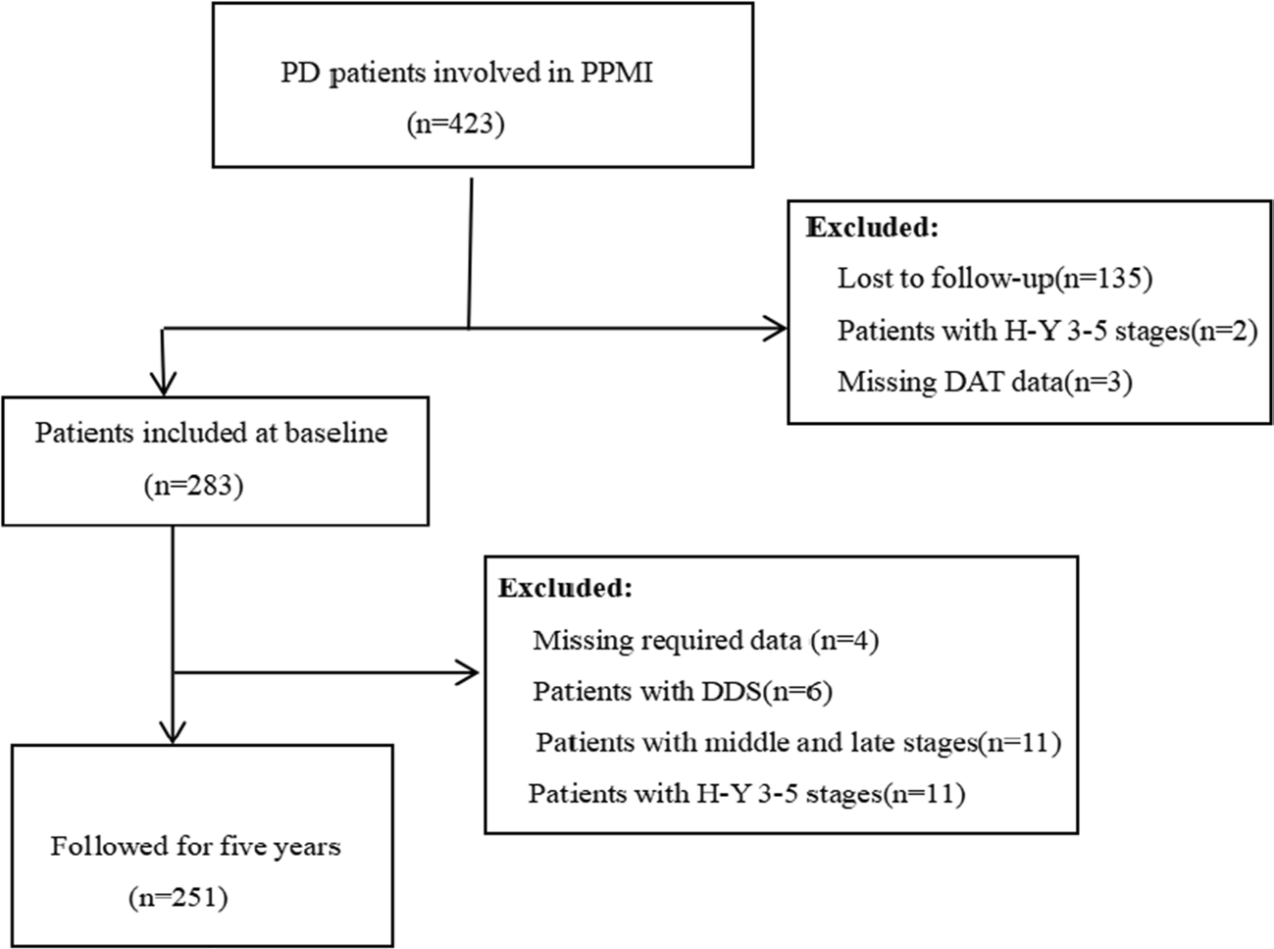
Flow chart of the selection of research objects

In this study, the patients were recruited between 2010 and 2015. At baseline, participants must be over 30 years of age with asymmetric resting tremor, asymmetric bradykinesia, or two of the following three signs: bradykinesia, resting tremor, and rigidity. Participants were not treated with medications for Parkinson’s disease within 60 days of the baseline visit [14]. Details of the PD inclusion/exclusion criteria adopted by the PPMI can be found at http://www.ppmi-info.org. This article was downloaded from http://www.ppmi-info.org. on May 30, 2022. At the time of data collection, a total of 423 patients with PD were included in the baseline study, and 283 patients had been followed up once a year for a total of 5 years. In PD patients, DDS onset was used as an outcome variable; patients were free of DDS at baseline, and the date of first onset of DDS during the follow-up period was recorded. After the exclusion of patients with DDS at baseline and those who had missing required data, the final study sample consists of 251 patients with early PD, and the flow chart of subject selection is shown in Fig. 1.

### Clinical characteristics and evaluation

The Movement Disorder Society-Unified Parkinson’s Disease Rating Scale (MDS-UPDRS) [15] was used to assess the subjects’ motor and nonmotor symptoms, and the Montreal Cognitive Assessment (MoCA) [16] was used to assess the cognitive status of the patients. The MDS-UPDRS 1.6 was used to evaluate DDS, and the MDS-UPDRS 1.3 and 1.4 were used to assess the depression and anxiety of the patients. Motor severity was assessed with the MDS-UPDRS III score. Rapid eye movement sleep behavior disorder (RBD) was used to assess the patient’s sleep. Impulse control disorder (ICD) and other compulsive behaviors were assessed by the Questionnaire for Impulsive-Compulsive Disorders in Parkinson’s Disease (QUIP). For patients taking DRT during follow-up, both motor and nonmotor symptom scores were assessed during the “On” period. Levodopa, dopamine agonists, amantadine, or monoamine oxidase B inhibitors are defined as DRT [17]. The Hoehn-Yahr (H-Y) stage is mainly used to evaluate the severity of PD patients and is divided into stages 1–5, in which stages 1–2 are defined as early stages and stages 2.5–5 are defined as middle and late stages [18].

### DAT imaging

All patients were enrolled with DAT-SPECT to rule out other conditions. DaTscan SPECT imaging was performed at various PPMI sites, and data analysis was performed by experienced nuclear medicine experts at a central imaging core. This imaging technique delineates the dopamine cells in regions of interest within the left and right striatum, caudate nucleus, and putamen by analyzing their DAT uptake values. DAT uptake rates in the caudate and putamen were calculated as the count density in the caudate and putamen divided by the count density in the occipital cortex as the reference region.

### Statistical analysis

GraphPad Prism version 8.0 (GraphPad Software, Inc., San Diego, CA, USA) software was used for graphing, and SPSS 26.0 (IBM, USA) statistical software was used for statistical analysis. Normally distributed data are expressed as the mean ± standard deviation ($\overline{x }$±*s*), and the independent samples *t* test was used for intergroup comparisons. Nonnormally distributed data are expressed as the median [*M* (*Q*1, *Q*3)], and the Mann‒Whitney *U* test was used for intergroup comparisons. Categorical data are expressed as a frequency (percentage), and the chi-square test was used for intergroup comparisons. The variables with statistically significant differences in this study were included in the univariate Cox regression analysis, while those with *P* ≤ 0.1 were included in the multivariate Cox regression analysis to determine risk factors. A paired sample t test was used to compare DAT uptake values of early PD patients before and after DDS. A Kaplan‒Meier (*K*-*M*) survival curve was used to describe the time-dependent trend of DDS, and a value of* P* < 0.05 was considered to indicate a statistically significant difference.

## Results

The demographic and clinical characteristics of early PD patients with DDS (*n* = 6) and without DDS (*n* = 277) at baseline are shown in Table 1. In our study, the incidence of DDS was approximately 2.2%. Depression (*P* = 0.019), stereotypic behavior (*P* = 0.003), RBD (*P* = 0.013), and UPDRS I (*P* = 0.006) scores were higher in DDS patients than in early PD patients without DDS. There were no significant differences in age, years of education, course of disease, sex, age of onset, UPDRS II score, UPDRS III score, total UPDRS score, H-Y stage, anxiety, fatigue, nonmotor symptom scores, and DAT uptake between the two groups.

**Table 1:** Baseline demographic and clinical characteristics of early PD patients with and without DDS

Variable	PD without DDS (*n* = 277)	PD with DDS (*n* = 6)	*t*/*z*/χ^2^	*P value*
Age (years)	61.8 (54.5, 68.6)	54.4 (47.7, 63.3)	− 1.608 ^a^	0.108
Education (years)	16.0 (14.0, 18.0)	16.0 (15.0, 19.3)	− 0.934 ^a^	0.350
Course of disease (years)	4.1 (2.5, 7.7)	8.9 (3.7, 12.9)	− 1.210 ^a^	0.226
Sex (male/female)	186/91	3/3	0.778	0.378
Age of onset (years)	60.0 (52.7, 66.6)	53.4 (46.8, 61.8)	− 1.477 ^a^	0.148
UPDRS I	5.0 (2.0, 7.0)	10.0 (7.0, 12.5)	− 2.734 ^a^	0.006
UPDRS II	4.0 (2.0, 8.0)	3 (1.8, 11.8)	− 0.350 ^a^	0.726
UPDRS III	19.0 (14.0, 25.0)	16.0 (12.8, 21.8)	− 0.775 ^a^	0.439
Total UPDRS	29.0 (21.0, 39.0)	28.5 (24.3, 42.5)	− 0.298 ^a^	0.766
H-Y stage [*n* (%)]				
Stage 1 Stage 2	138 (49.8)	3 (50.0)	0.000	0.993
	139 (50.2)	3 (50.0)		
Depression [*n* (%)]	68 (24.5)	4 (66.7)	5.492	0.019
Anxiety [*n* (%)]	101 (36.5)	4 (66.7)	2.296	0.130
Pathological gambling [*n* (%)]	2 (0.7)	0 (0)	0.044	0.835
Hypersexuality [*n* (%)]	7 (2.5)	0 (0)	0.155	0.693
Compulsive shopping [*n* (%)]	8 (2.9)	0 (0)	0.178	0.673
Compulsive eating [*n* (%)]	26 (9.4)	0 (0)	0.620	0.431
Pathological hobby [*n* (%)]	24 (8.7)	0 (0)	0.568	0.451
Punding [*n* (%)]	14 (5.1)	2 (33.3)	8.805	0.003
Walking or driving [*n* (%)]	3 (1.1)	0 (0)	0.066	0.798
RBD [*n* (%)]	95 (34.3)	5 (83.3)	6.180	0.013
MoCA score	28.0 (26.0, 29.0)	28.5 (23.0, 30.0)	− 0.380 ^a^	0.704
DAT uptake				
Mean caudate	2.0 ± 0.5	2.0 ± 0.9	− 0.497 ^b^	0.619
Mean putamen	0.79 (0.6, 1.0)	0.76 (0. 5,1.2)	− 0.076 ^a^	0.940

Abbreviations: ^a^The z score was obtained by the Mann‒Whitney *U* test. ^b^The *t* value was obtained by the independent samples *t test*. The remaining data represent the χ^2^ values obtained by the chi-square test. *RBD*, rapid eye movement sleep behavior disorder; *H-Y*, Hoehn-Yahr; *MDS-UPDRS*, International Parkinson’s Disease and Movement Disorders Association Unified Parkinson’s Disease Rating Scale; *MoCA*, Montreal Cognitive Assessment; *ESS*, Epworth sleep; *DAT*, dopamine transporter

### Independent predictors of DDS in early PD patients

**Fig. 2:**
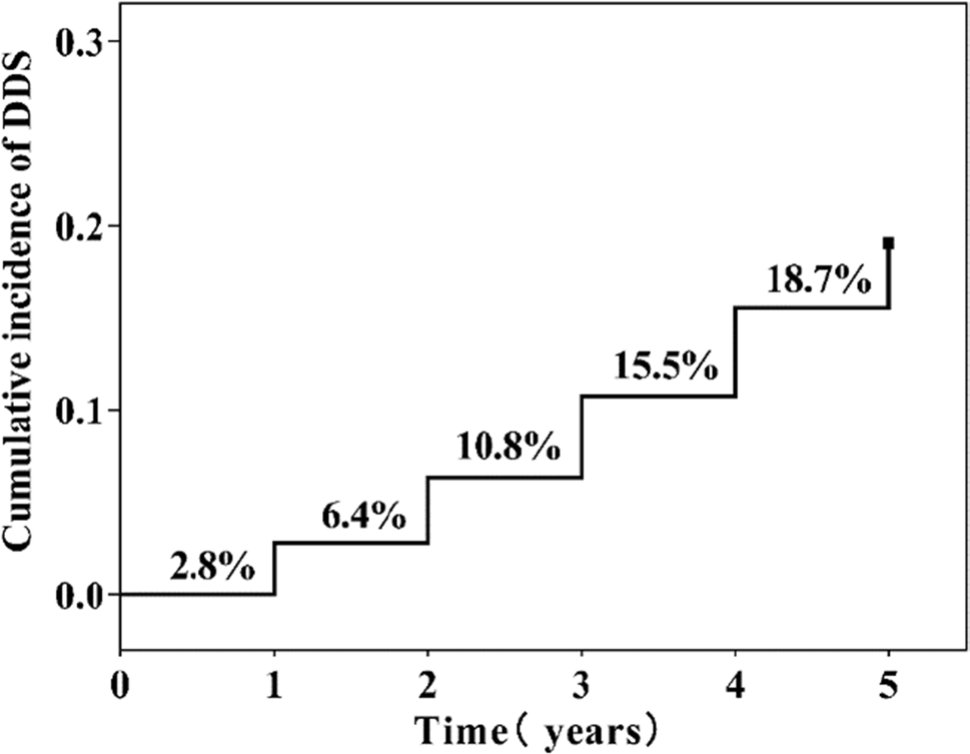
Kaplan‒Meier estimates showing the cumulative incidence of DDS

A total of 251 patients completed the full follow-up study, of whom 47 patients developed DDS during follow-up. In our study, the cumulative incidence of DDS increases over time, as shown in Fig. 2. To explore the risk factors for the occurrence of early PD, we included age, sex, drug, and variables with* P* ≤ 0.1 in the univariate Cox regression analysis in the multivariate Cox regression analysis. The regression analysis used the forward stepwise variable selection method to adjust the influence of confounding factors on the occurrence of DDS. After adjusting for related factors, hyperactivity (HR = 3.088, 95% CI 1.416~6.732,* P* = 0.005), compulsive eating (HR = 3.299, 95% CI 1.665~6.534, *P* = 0.001), compulsive shopping (HR = 3.899, 95% CI 1.769~8.593, *P* = 0.001), anxiety (HR = 4.018, 95% CI 2.136~7.559, *P* < 0.01), and H-Y stage 1 (HR = 0.278, 95% CI 0.52~0.509, *P* < 0.01) are identified as independent risk factors for DDS in early PD (*P* < 0.05), as shown in Table 2. This combined model produced an acceptable goodness of fit (χ^2^ = 59.024; *df* = 5;* P* < 0.001).

**Table 2:** Cox regression analysis results of potential predictors of DDS

Variable	Univariable analysis		Multivariable analysis	
	HR (95% CI)	*P value*	HR (95% CI)	*P value*
Age	0.975 (0.947~1.003)	0.082	NA	NA
Sex	1.412 (0.784~2.543)	0.250		
Age of onset	0.975 (0.948~1.003)	0.075	NA	NA
UPDRS I score	1.072 (1.031~1.115)	0.001	NA	NA
UPDRS III score	0.970 (0.944~0.996)	0.025	NA	NA
H-Y stage	0.292 (0.163~0.524)	< 0.01	0.278 (0.152~0.509)	< 0.01
Pathological gambling	3.616 (0.877~14.909)	0.075	NA	NA
Hypersexuality	4.369 (2.169~8.799)	< 0.01	3.088 (1.416~6.732)	0.005
Compulsive shopping	4.073 (1.901~8.727)	< 0.01	3.899 (1.769~8.593)	0.001
Compulsive eating	4.738 (2.533~8.861)	< 0.01	3.299 (1.665~6.534)	0.001
Pathological hobbies	2.661 (1.323~5.353)	0.006	NA	NA
Punding	2.443 (0.876~6.813)	0.088	NA	NA
Walking or driving	2.496 (0.344~18.102)	0.365		
Hallucinations and psychosis	1.803 (0.872~3.728)	0.112		
Depression	2.281 (1.286~4.046)	0.005	NA	NA
Anxiety	2.486 (1.388~4.453)	0.002	4.018 (2.136~7.559)	< 0.01
RBD	2.135 (1.178~3.872)	0.012	NA	NA
Medication				
Untreated	NA	0.094	NA	NA
Levodopa	0.788 (0.173~3.597)	0.758		
Dopamine agonist	2.746 (0.570~13.223)	0.208		
Other	1.469 (0.207~10.426)	0.701		
Levodopa + other	0.282 (0.040~2.000)	0.205		
Levodopa + dopamine agonist	1.430 (0.313~6.528)	0.644		
Dopamine agonist + other	1.566 (0.287~8.553)	0.604		
Levodopa + dopamine agonist + other	1.719 (0.376~7.846)	0.485		

*MDS-UPDRS*, International Parkinson’s Disease and Movement Disorders Association Unified Parkinson’s Disease Rating Scale; *H–Y stage*, Hoehn-Yahr stage; *RBD*, rapid eye movement sleep behavior disorder; *NA*, not applicable/not available; *CI*, confidence interval

### Kaplan–Meier survival curve analysis

**Fig. 3:**
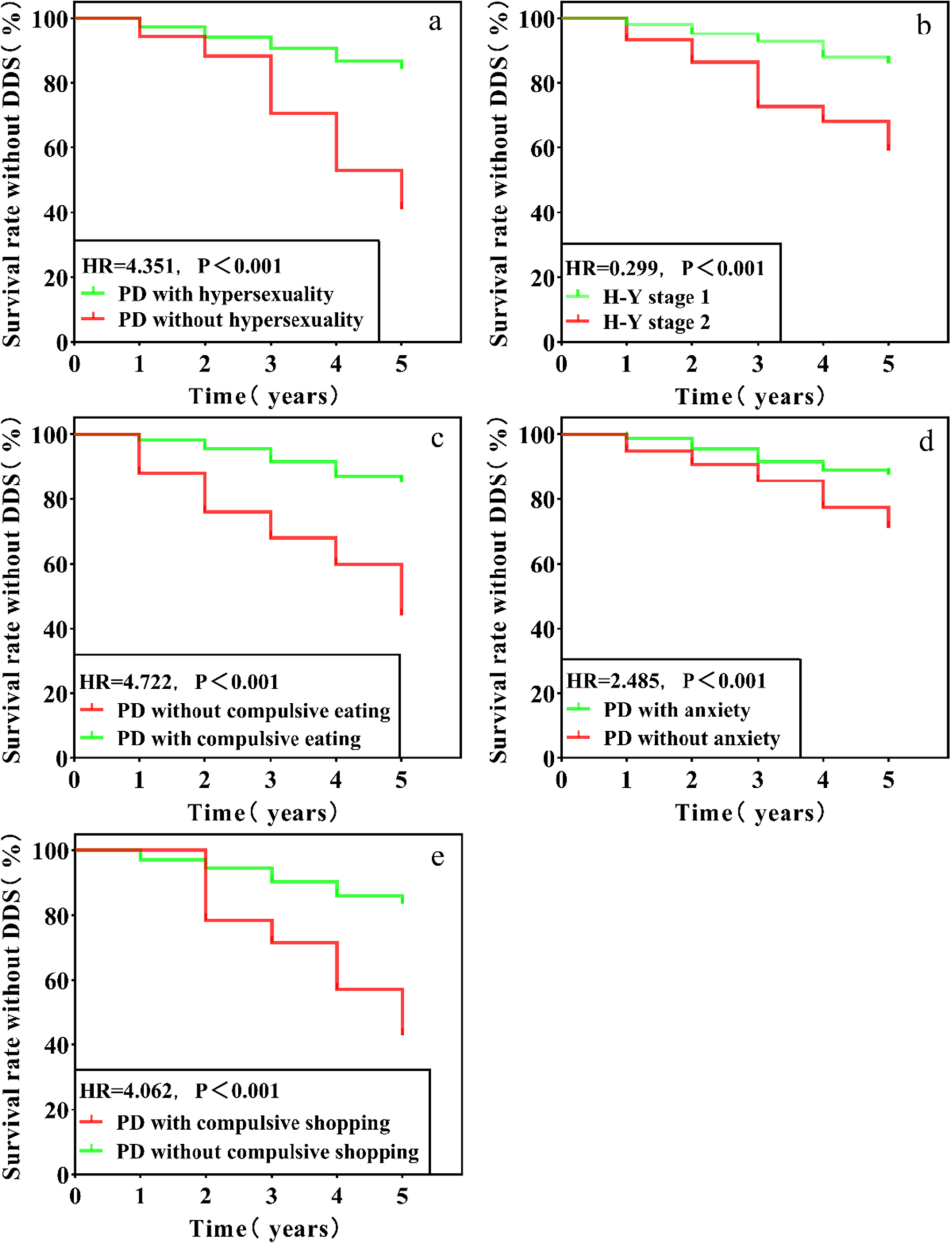
**a**–**e** Kaplan‒Meier survival estimation risk of DDS in early PD patients with hypersexuality, compulsive eating, compulsive shopping, anxiety, and H-Y stage 1

The comparison of PD patients with and without hypersexuality, compulsive eating, compulsive shopping, and anxiety showed that for those with these behaviors, there was a higher risk of developing DDS during follow-up (*P* < 0.05); furthermore, the lower the H-Y stage is during follow-up, the higher the risk of DDS (*P* < 0.05), as shown in Fig. 3a–e.

To further explore why H-Y stage 1 predicts the occurrence of DDS, we compared the clinical features of patients with H-Y stage 1 and stage 2 after follow-up and showed that the age, age of onset, UPDRSIII and total score, and mean uptake of caudate and putamen DAT in H-Y stage 1 were significantly lower than those in stage 2 (*P* < 0.05). However, there was no significant difference in disease course, sex, family history, depression, and ICD between H-Y stage 1 and 2 patients (*P* > 0.05) (Table 3).

**Table 3:** Comparison of characteristic data of H-Y stage 1 and 2 patients after follow-up

Variable	H-Y stage1 (*n* = 44)	H-Y stage2 (*n* = 207)	*t*/*z*/c^2^	*P value*
Age (years)	57.5 ± 9.7	61.5 ± 9.5	− 2.5 ^a^	0.013
Age of onset (years)	56.0 ± 9.9	59.3 ± 9.8	− 2.028 ^a^	0.044
Sex (male/female)	26/18	146/61	2.202 ^b^	0.138
Anxiety	13 (29.5)	84 (40.6)	1.863 ^b^	0.172
Depression	12 (27.3)	61 (29.5)	0.085 ^b^	0.771
Total UPDRS score	23.0 (22.25,31.5)	48.0 (34.3,62.5)	− 7.685	< 0.01
UPDRS III score	11.0 (9.25,13.0)	23.0 (14.0,32.5)	− 6.495	< 0.01
Family history of PD [n(%)]			2.242 ^b^	0.326
First-degree family member w/PD	4 (9.1)	33 (15.9)		
Non-1st degree family member w/PD	7 (15.9)	21 (10.1)		
No family member w/PD	33 (75)	153 (73.9)		

Abbreviations: ^a^The *t* value was obtained by the independent samples *t test*. ^b^The χ^2^ values obtained by the chi-square test; the remaining data represent the test *z* score obtained by the Mann-Whitney U test; *H-Y*, Hoehn-Yahr

### Comparison of DAT uptake rates before and after DDS in patients with early PD

**Fig. 4:**
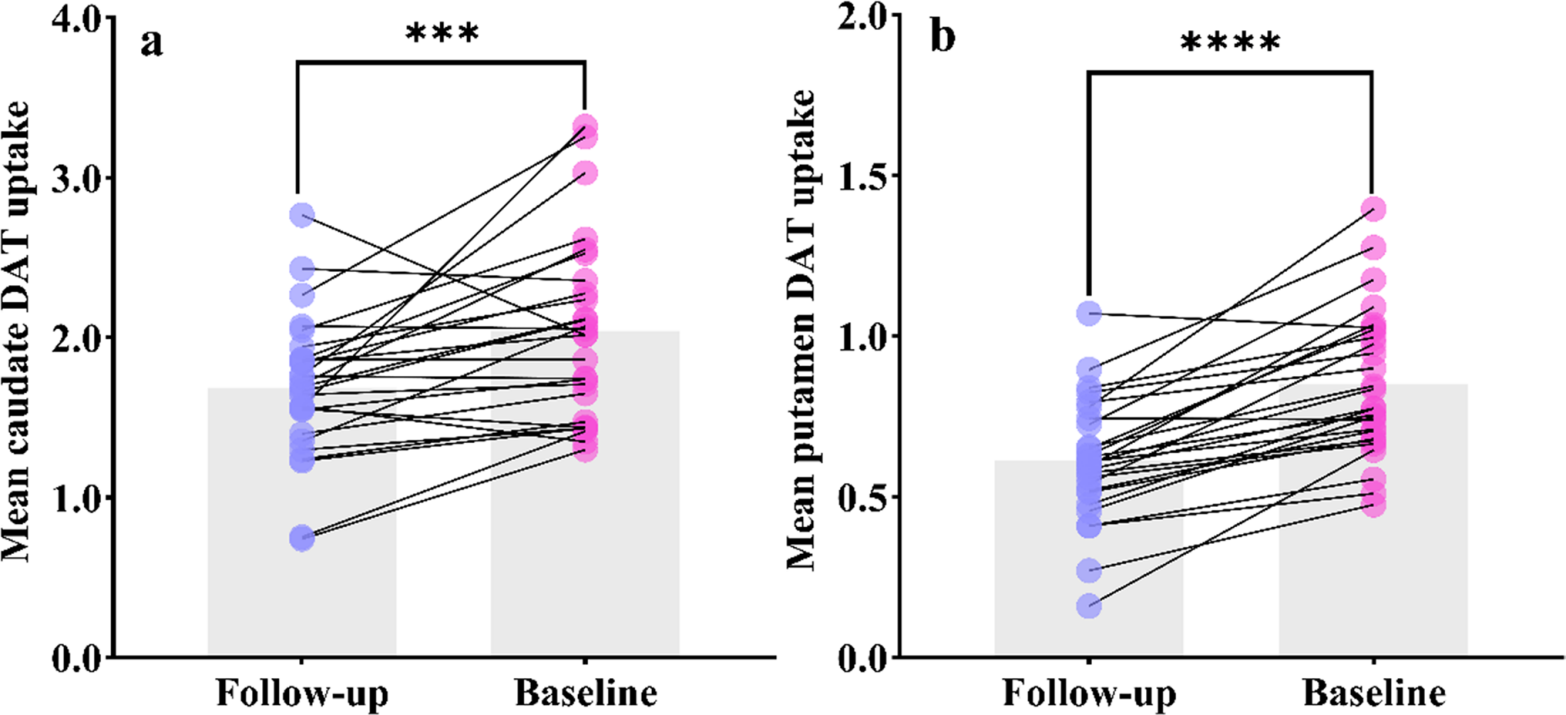
**a**, **b** Changes in the average uptake of DAT for the caudate and putamen before and after DDS in patients with early PD (***P < 0.001, ****P < 0.0001). DAT: Dopamine transporter

A total of 47 patients developed DDS at the end of follow-up, of whom only 27 had complete data on DAT uptake. The DAT uptake values before and after follow-up were used as a paired *t* test. It is found that in both the caudate nucleus and the putamen, the baseline DAT uptake values of these 27 patients were higher than at the end of the follow-up, as shown in Fig. 4.

### The relationship between survival without DDS and medicine

**Fig. 5:**
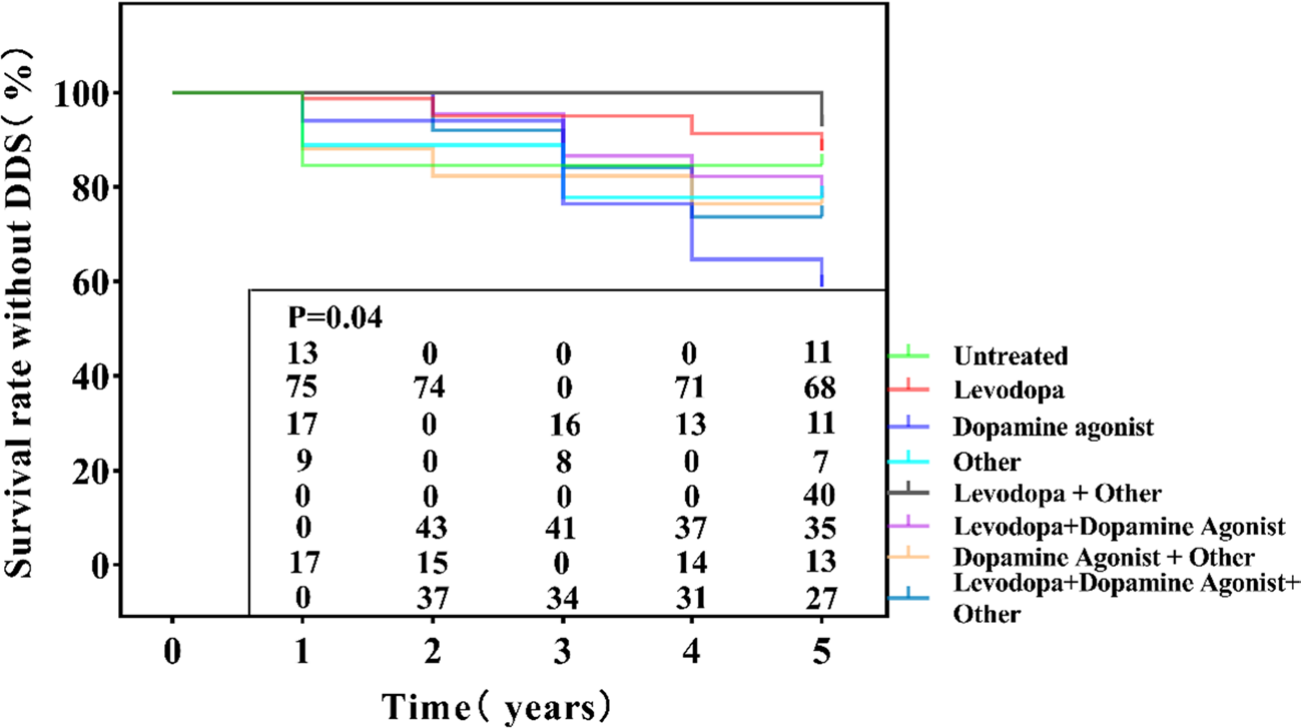
The relationship between survival without DDS and drug use. HR = hazard ratio

Survival curve analysis was performed with respect to the drug use of patients. The results showed that patients taking dopamine receptor agonists had the highest risk of DDS, while patients taking levodopa and other drugs had the lowest risk of DDS (Fig. 5).

## Discussion

Our findings are mainly in the following aspects. First, the incidence of DDS was approximately 2.2%, and the cumulative incidence of DDS gradually increased with the extension of follow-up time. Second, hypersexuality, compulsive eating, compulsive shopping, anxiety and lower H-Y stage were independent risk factors for the development of DDS. Finally, DAT uptake values correlate with DDS occurrence. Patients taking dopamine agonists had a higher risk of developing DDS than patients taking levodopa.

Previous studies have shown a wide variation in the prevalence of DDS, ranging from 2.2 to 10.4% [5, 6, 8, 19, 20]. Accumulating evidence suggests that DDS in PD patients is the result of a history of dopaminergic drug use, genetic predisposition, history of psychiatric disorders, and impulsive personality [9, 12]. The reason why the incidence of DDS in this study was slightly lower than that in previous studies may be related to the following aspects: first, unlike this study, most patients in previous studies received dopaminergic drug treatment [6, 20]. Second, the patients included in this study were mostly Caucasian and had a shorter disease duration, whereas the patients in the study by Tsai et al. were Asian and had a longer disease duration [19, 21, 22]. Finally, it may be related to the early PD of the patients in this study. At present, there are few and different conclusions about the incidence of DDS at home and abroad, and the prevalence of DDS still needs further research.

In the past 10 years, there has been very little research on risk factors for the development of DDS. In our study, patients with impulse control disorder (ICD)-related symptoms (hypersexuality, compulsive eating, and compulsive shopping) were more likely to develop DDS, which is consistent with the findings of Cilia et al. [12]. Previous studies have confirmed that the occurrence of ICD is related to the overstimulation of dopamine receptors in the mesolimbic pathway [23], and the occurrence of DDS is often accompanied by ICD [24, 25]. Therefore, our findings suggest that patients with ICD are prone to DDS, possibly due to the similar pathophysiological mechanisms of DDS and ICD [26]. In addition, in the study by Tanaka et al., patients taking levodopa were more likely to develop DDS than dopamine agonists [9]. However, our study found that patients taking dopamine agonists had a higher risk of developing DDS. Because ICD-related symptoms are an independent risk factor for DDS in the patients in this study, some studies have confirmed that the occurrence of ICD is mostly related to dopamine agonists [27]. Therefore, the reason why our results are inconsistent with previous studies may be that dopamine agonists are a confounding factor in the occurrence of DDS by affecting ICD and thus DDS.

Previous studies have established a link between depression and the occurrence of DDS [11]. However, we did not conclude that depression could independently predict the occurrence of DDS in our study. Differences in follow-up time, assessment tools, genetic background, and study population may contribute to this difference. Notably, our study found anxiety to be an independent predictor of DDS development. To our knowledge, the occurrence of DDS is associated with dopamine replacement therapy, especially levodopa. In view of the results of this study, we can make the following explanations. First, compared with PD patients, DDS patients are more prone to mood disorders, which may be related to the sensitization of the ventral striatum caused by long-term high-dose dopaminergic drugs [28]. Second, levodopa has traditionally been known to improve mood [29]. However, studies have confirmed that levodopa not only does not improve mood [30] but also aggravates mood disorders [31]. At present, the neurobiological mechanism of levodopa on the occurrence of anxiety is still unclear. Recent studies in animal models of PD suggest that levodopa may interfere with the function of the norepinephrine and serotonin systems in emotion-related brain structures [32, 33], thereby inducing the development of anxiety and depression. Finally, mood disorders in turn increase vulnerability to substance abuse risk [34]. Thus, our results suggest that the intrinsic disease process by which PD predisposes patients to mood disorders may affect dopaminergic neurotransmission, thereby increasing susceptibility to DDS. In addition, two studies have demonstrated that antipsychotics can improve some of the symptoms of DDS [35, 36], indicating that DDS and mood disorders interact and influence each other.

In addition, a novel finding of our study is that the H-Y stage is associated with the development of DDS, and interestingly, patients with H-Y stage 1 are more likely to develop DDS than PD patients with H-Y stage 2. In the study of Cilia et al. [12], there was no significant difference in H-Y staging between the two groups, which may be related to the off-stage staging of the study. To further analyze the reason why the incidence of DDS in H-Y stage 1 is higher than that in stage 2, we compared the clinical characteristics of patients with H-Y stages 1 and 2 after follow-up and found that the age, age of onset, UPDRSIII score, and UPDRS total score of PD patients with H-Y stage 1 were significantly lower than those of PD patients with stage 2. Our results can be interpreted as follows. First, many studies have confirmed that PD patients with younger onset age are more likely to develop DDS [10–12]. This may be related to the fact that PD patients with younger onset age can establish comprehensive Parkinson’s disease clinical manifestations faster, worsen the treatment effect of levodopa, have earlier side effects, and are more prone to motor fluctuations [37]. In addition, it has been confirmed that age may significantly mediate vulnerability to drug addiction [38]. Although PD patients are mostly middle-aged and elderly, earlier exposure to addictive drugs in patients with a younger age of onset affects the subsequent development and severity of substance dependence. This result may be related to the lower age and age of onset of H-Y stage 1 patients. Second, patients with lower stages have milder symptoms and may have higher requirements for their own mobility.

Finally, many studies have confirmed that ICD is related to the function of DAT [39–42], but the relationship between the occurrence of DDS and DAT uptake is still unclear. We analyzed DAT uptake values before and after DDS in patients with early PD and found that mean DAT uptake values in the caudate and putamen were significantly lower in patients with DDS after follow-up compared with baseline. It can be speculated that the occurrence of DDS may be related to the low uptake value of DAT. Due to the lack of data on DAT uptake values after follow-up in this study, we could not explore whether DAT uptake values could predict the occurrence of DDS. In the future, the sample size can be increased to further explore the neurobiological mechanism of DAT function affecting the occurrence of DDS.

The strengths of this study include the long-term follow-up, relatively large sample size, and new-onset PD cohort study. Although some missing data in the dataset may affect the results, the regression model constructed in our statistical analysis fit well. However, this study also has certain limitations. First, the diagnosis of the above factors is only based on scale evaluation, without objective quantitative index description. Second, the data related to DAT function in this study were missing a lot, and the relationship between DAT intake value and the risk of DDS was not explored. In future research, the DAT data of PD patients will be examined, and the relationship between DAT uptake and DDS will be further explored.

## Conclusion

Our study found that hypersexuality, compulsive eating, compulsive shopping, anxiety, and lower H-Y stage increase the risk of DDS in patients with early PD, and these factors may help identify early patients who are more likely to develop DDS and help minimize consequences. Additionally, for PD patients with an earlier onset age, levodopa therapy should be delayed as much as possible to reduce the risk of DDS in patients with early-onset PD. In addition, before treating PD patients, clinicians should also assess whether the patients have risk factors for DDS and intervene in time to improve the patients’ quality of life.

## Data Availability

All data reported in this article are available in the PPMI database (https://www.ppmi-info.org/.).

## References

[1] Bloem BR, Okun MS, Klein C (2021). Parkinson's disease. Lancet (London, England).

[2] Tolosa E, Garrido A, Scholz SW, Poewe W (2021). Challenges in the diagnosis of Parkinson's disease. The Lancet Neurol.

[3] Reich SG, Savitt JM (2019). Parkinson's Disease. Med Clin North Am.

[4] Giovannoni G, O'Sullivan JD, Turner K, Manson AJ, Lees AJ (2000). Hedonistic homeostatic dysregulation in patients with Parkinson's disease on dopamine replacement therapies. J Neurol, Neurosurg, Psychiatry.

[5] Xu T, Cao L, Long W, Zhao G (2021). Validation of the Chinese version of the questionnaire for impulsive-compulsive disorders in Parkinson's disease. Front Neurol.

[6] Rodriguez-Blazquez C, Schrag A, Rizos A, Chaudhuri KR, Martinez-Martin P, Weintraub D (2021). Prevalence of Non-motor symptoms and non-motor fluctuations in Parkinson's disease using the MDS-NMS. Mov Disord Clin Pract.

[7] O'Sullivan SS, Evans AH, Lees AJ (2009). Dopamine dysregulation syndrome: an overview of its epidemiology, mechanisms and management. CNS drugs.

[8] Gulunay A, Cakmakli GY, Yon MI, Ulusoy EK, Karakoc M (2020). Frequency of non-motor symptoms and their impact on the quality of life in patients with Parkinson's disease: a prospective descriptive case series. Psychogeriatrics.

[9] Tanaka K, Wada-Isoe K, Nakashita S, Yamamoto M, Nakashima K (2013). Impulsive compulsive behaviors in Japanese Parkinson's disease patients and utility of the Japanese version of the questionnaire for impulsive-compulsive disorders in Parkinson's disease. J Neurol Sci.

[10] Warren N, O'Gorman C, Lehn A, Siskind D (2017). Dopamine dysregulation syndrome in Parkinson's disease: a systematic review of published cases. J Neurol, Neurosurg, Psychiatry.

[11] Evans AH, Lawrence AD, Potts J, Appel S, Lees AJ (2005). Factors influencing susceptibility to compulsive dopaminergic drug use in Parkinson disease. Neurology.

[12] Cilia R, Siri C, Canesi M, Zecchinelli AL, De Gaspari D, Natuzzi F, Tesei S, Meucci N, Mariani CB, Sacilotto G, Zini M, Ruffmann C, Pezzoli G (2014). Dopamine dysregulation syndrome in Parkinson's disease: from clinical and neuropsychological characterisation to management and long-term outcome. J Neurol, Neurosurg Psychiatry.

[13] Parkinson Progression Marker Initiative (2011). The Parkinson progression marker initiative (PPMI). Progress Neurobiol.

[14] Schrag A, Siddiqui UF, Anastasiou Z, Weintraub D, Schott JM (2017). Clinical variables and biomarkers in prediction of cognitive impairment in patients with newly diagnosed Parkinson's disease: a cohort study. The Lancet Neurol.

[15] Goetz CG, Tilley BC, Shaftman SR, Stebbins GT, Fahn S, Martinez-Martin P, Poewe W, Sampaio C, Stern MB, Dodel R, Dubois B, Holloway R, Jankovic J, Kulisevsky J, Lang AE, Lees A, Leurgans S, LeWitt PA, Nyenhuis D, Olanow CW, Rascol O, Schrag A, Teresi JA, van Hilten JJ, LaPelle N (2008). Movement disorder society-sponsored revision of the Unified Parkinson's disease rating scale (MDS-UPDRS): scale presentation and clinimetric testing results. Mov Disord.

[16] Dalrymple-Alford JC, MacAskill MR, Nakas CT, Livingston L, Graham C, Crucian GP, Melzer TR, Kirwan J, Keenan R, Wells S, Porter RJ, Watts R, Anderson TJ (2010). The MoCA: well-suited screen for cognitive impairment in Parkinson disease. Neurology.

[17] de la Riva P, Smith K, Xie SX, Weintraub D (2014). Course of psychiatric symptoms and global cognition in early Parkinson disease. Neurology.

[18] Goetz CG, Poewe W, Rascol O, Sampaio C, Stebbins GT, Counsell C, Giladi N, Holloway RG, Moore CG, Wenning GK, Yahr MD, Seidl L (2004). Movement disorder society task force report on the Hoehn and Yahr staging scale: status and recommendations. Mov Disord.

[19] Barbosa P, Djamshidian A, Lees AJ, Warner TT (2018). The outcome of dopamine dysregulation syndrome in Parkinson's disease: a retrospective postmortem study. Mov Disord Clin Pract.

[20] El Otmani H, Mouni FZ, Abdulhakeem Z, Attar Z, Rashad L, Saali I, El Moutawakil B, Rafai MA, Slassi I, Nadifi S (2019). Impulse control disorders in Parkinson disease: a cross-sectional study in Morocco. Revue Neurologique.

[21] Maeda T, Shimo Y, Chiu SW, Yamaguchi T, Kashihara K, Tsuboi Y, Nomoto M, Hattori N, Watanabe H, Saiki H (2017). Clinical manifestations of nonmotor symptoms in 1021 Japanese Parkinson's disease patients from 35 medical centers. Parkinsonism Relat Disord.

[22] Tsai ST, Hung HY, Hsieh TC, Lin SH, Lin SZ, Chen SY (2013). Long-term outcome of young onset Parkinson's disease after subthalamic stimulation–a cross-sectional study. Clin Neurol Neurosurg.

[23] Weintraub D, Mamikonyan E (2019). Impulse control disorders in Parkinson's disease. Am J Psychiatry.

[24] Wise RA, Rompre PP (1989). Brain dopamine and reward. Annu Rev Psychol.

[25] Tanwani P, Fernie BA, Nikčević AV, Spada MM (2015). A systematic review of treatments for impulse control disorders and related behaviours in Parkinson's disease. Psychiatry Res.

[26] Evans AH, Strafella AP, Weintraub D, Stacy M (2009). Impulsive and compulsive behaviors in Parkinson's disease. Mov Disord.

[27] Fenu S, Wardas J, Morelli M (2009). Impulse control disorders and dopamine dysregulation syndrome associated with dopamine agonist therapy in Parkinson's disease. Behav Pharmacol.

[28] Eskow Jaunarajs KL, Angoa-Perez M, Kuhn DM, Bishop C (2011). Potential mechanisms underlying anxiety and depression in Parkinson's disease: consequences of l-DOPA treatment. Neurosci Biobehav Rev.

[29] Yahr MD, Duvoisin RC, Schear MJ, Barrett RE, Hoehn MM (1969). Treatment of parkinsonism with levodopa. Arch Neurol.

[30] Kim HJ, Park SY, Cho YJ, Hong KS, Cho JY, Seo SY, Lee DH, Jeon BS (2009). Nonmotor symptoms in de novo Parkinson disease before and after dopaminergic treatment. J Neurol Sci.

[31] Choi C, Sohn YH, Lee JH, Kim J (2000). The effect of long-term levodopa therapy on depression level in de novo patients with Parkinson's disease. J Neurol Sci.

[32] Eskow Jaunarajs KL, Dupre KB, Ostock CY, Button T, Deak T, Bishop C (2010). Behavioral and neurochemical effects of chronic L-DOPA treatment on nonmotor sequelae in the hemiparkinsonian rat. Behav Pharmacol.

[33] Navailles S, Bioulac B, Gross C, De Deurwaerdère P (2010). Serotonergic neurons mediate ectopic release of dopamine induced by L-DOPA in a rat model of Parkinson's disease. Neurobiol Dis.

[34] Volkow ND (2004). The reality of comorbidity: depression and drug abuse. Biol Psychiatry.

[35] Vargas AP, Cardoso FEC (2018). Impulse control and related disorders in Parkinson's disease. Arq de Neuro-Psiquiatr.

[36] Gerschlager W, Bloem BR (2009). Managing pathological gambling in Parkinson's disease with enteral levodopa/carbidopa infusions. Mov Disord.

[37] Giovannini P, Piccolo I, Genitrini S, Soliveri P, Girotti F, Geminiani G, Scigliano G, Caraceni T (1991). Early-onset Parkinson's disease. Mov Disord.

[38] Chambers RA, Taylor JR, Potenza MN (2003). Developmental neurocircuitry of motivation in adolescence: a critical period of addiction vulnerability. Am J Psychiatry.

[39] Vriend C, Nordbeck AH, Booij J, van der Werf YD, Pattij T, Voorn P, Raijmakers P, Foncke EM, van de Giessen E, Berendse HW, van den Heuvel OA (2014). Reduced dopamine transporter binding predates impulse control disorders in Parkinson's disease. Mov Disord.

[40] Smith KM, Xie SX, Weintraub D (2016). Incident impulse control disorder symptoms and dopamine transporter imaging in Parkinson disease. J Neurol, Neurosurg, Psychiatry.

[41] Cilia R, Ko JH, Cho SS, van Eimeren T, Marotta G, Pellecchia G, Pezzoli G, Antonini A, Strafella AP (2010). Reduced dopamine transporter density in the ventral striatum of patients with Parkinson's disease and pathological gambling. Neurobiol Dis.

[42] Voon V, Rizos A, Chakravartty R, Mulholland N, Robinson S, Howell NA, Harrison N, Vivian G, Ray Chaudhuri K (2014). Impulse control disorders in Parkinson's disease: decreased striatal dopamine transporter levels. J Neurol, Neurosurg, Psychiatry.

